# Assessment of Myocardial Injury Size Metrics Using Carotid Pressure Waveform: Proof‐of‐Concept in Coronary Occlusion/Reperfusion Rat Model

**DOI:** 10.1096/fj.202502111R

**Published:** 2025-09-06

**Authors:** Jiajun Li, Rashid Alavi, Wangde Dai, Ray V. Matthews, Robert A. Kloner, Niema M. Pahlevan

**Affiliations:** ^1^ Department of Aerospace and Mechanical Engineering University of Southern California Los Angeles California USA; ^2^ Department of Medical Engineering California Institute of Technology Pasadena California USA; ^3^ Cardiovascular Research Institute Huntington Medical Research Institutes Pasadena California USA; ^4^ Division of Cardiovascular Medicine, Keck School of Medicine University of Southern California Los Angeles California USA; ^5^ Cardiac and Vascular Institute University of Southern California Los Angeles California USA

**Keywords:** acute myocardial infarction, arterial pressure waveform, cardiovascular intrinsic frequency, myocardial injury sizes, physics‐based machine learning

## Abstract

Myocardial infarction (MI) is a leading cause of death worldwide and the most common precursor to heart failure, even after initial treatment. Precise evaluation of myocardial injury is crucial for assessing interventions and improving outcomes. Extensive evidence from both preclinical models and clinical studies demonstrates that the extent and severity of myocardial injury (i.e., myocardial infarct size, ischemic risk zone, and no‐reflow area) are critical determinants of long‐term outcomes post‐MI. This study aims to assess whether carotid pressure waveforms, analyzed using an intrinsic frequency (IF)–machine learning (ML) approach, can accurately quantify myocardial injury sizes: myocardial infarct size, ischemic risk zone, and no‐reflow area. Acute MI was induced in *N* = 88 Sprague‐Dawley rats using a standard coronary occlusion/reperfusion model. MI‐injury sizes were obtained via histopathology. IF metrics were extracted from carotid pressure waveforms post‐MI. ML classifiers were developed using 66 rats and externally tested on 22 additional rats. Our best developed model for infarct size achieved an accuracy of 0.95 (specificity = 0.95, sensitivity = 0.96). For the ischemic risk zone, the best model showed an accuracy of 0.85 (specificity = 0.90, sensitivity = 0.80), and for the no‐reflow area, we reached an accuracy of 0.88 (specificity = 0.89, sensitivity = 0.86). To conclude, a hybrid physics‐based ML approach applied to carotid pressure waveforms successfully classified MI‐injury severity. As carotid pressure waveforms can be measured non‐invasively and remotely (e.g., via smartphones), this proof‐of‐concept preclinical study suggests a translational potential for post‐MI management, enabling timely interventions, improved patient monitoring, and mitigating adverse outcomes.

## Graphical abstract

In a coronary occlusion/reperfusion rat model, a single carotid pressure waveform was analyzed using a physics‐based approach to classify myocardial infarct size, ischemic risk zone, and no‐reflow area with high accuracy. Injury sizes were validated by histology. This proof‐of‐concept demonstrates the potential of carotid pressure waveforms for non‐invasive, instantaneous, and scalable assessment of myocardial injury severity following acute myocardial infarction, enabling timely interventions, improved patient monitoring, and mitigating adverse outcomes.

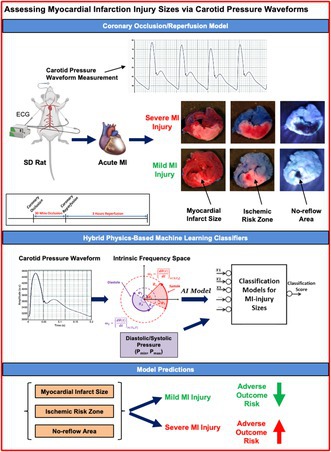

**Abbreviations**
AN: area of necrosis
A‐NR: no‐reflow area
AR: area of ischemic risk zone
AUC: area under the curve
CV: cross‐validation
DBP: diastolic blood pressure
DPP: diastolic peak pressure
IF: intrinsic frequency
KNN: K‐Nearest Neighbor
LV: left ventricle
MI: myocardial infarction
ML: machine learning
ROC: receiver operating characteristic
SBP: systolic blood pressure
SD: standard deviation
STFR: sparse time‐frequency representation
SVC: Support Vector Classifier

## 1 Introduction

Myocardial infarction (MI) is a leading cause of morbidity and mortality worldwide [1, 2] and remains the most common precursor to the development of heart failure [3]. Despite advances in early reperfusion therapy, a substantial proportion of MI survivors experience adverse ventricular remodeling and progressive systolic dysfunction, ultimately culminating in chronic heart failure [4]. Extensive evidence from both preclinical models and clinical studies demonstrates that the extent and severity of myocardial injury (i.e., myocardial infarct size, ischemic risk zone, and no‐reflow area [5, 6, 7, 8, 9]) are critical determinants of long‐term outcomes post‐MI. An observational study showed that assessing final infarct size 3 months after ST‐segment elevation MI (STEMI) provides strong, independent prognostic value beyond established risk factors, potentially improving risk stratification in STEMI patients [10]. In preclinical models, rats with larger infarctions consistently developed overt congestive heart failure, with elevated filling pressures, reduced cardiac output, and a minimal capacity to respond to pre‐ and afterload stresses [9]. Furthermore, clinical imaging studies have demonstrated that the presence of no‐reflow on myocardial contrast echocardiography (MCE) independently predicts both early and late congestive heart failure, as well as significant left ventricular dilation, regardless of infarct location or size of the risk zone [8].

Assessing MI injury sizes serves as a pivotal surrogate endpoint for gauging the efficacy of therapies and carries prognostic value for post‐MI complications such as heart failure and ventricular remodeling [4, 11, 12]. While traditional MI injury measurement techniques such as echocardiography (Echo) [13], nuclear magnetic resonance imaging (MRI) [14], computed tomography (CT) [15], single photon emission computed tomography (SPECT) [16], and positron emission tomography (PET) [17] offer avenues for estimating the MI injury sizes, they are encumbered by various limitations including radiation exposure, reliance on expert operators, prolonged procedural times, and high costs. Given these critical considerations, there exists an unmet need for non‐invasive, instantaneous, and cost‐effective techniques to assess myocardial infarct size, ischemic risk zone, and no‐reflow area.

During the progression of MI, LV contractile performance declines, and LV filling pressure rises, marking initial hemodynamic abnormalities [18]. According to previous preclinical and clinical studies, we hypothesize that a hybrid intrinsic frequency (IF)‐machine learning (ML) method can identify pathophysiological signatures of MI by assessing the MI injury sizes from arterial pressure waveforms [19, 20, 21, 22, 23]. The IF method computes dominant instantaneous frequencies during systole (*ω*_1_) and diastole (*ω*_2_), reflecting LV contractile function and arterial dynamics, respectively [19, 20, 21, 22, 24, 25]. Lower total frequency variation (Δ*ω* = *ω*_1_ − *ω*_2_) indicates optimal ventricular‐vascular coupling [19, 20, 26]. This method is calibration‐independent and has shown clinical relevance in providing insights into LV function, vascular dynamics, and LV‐arterial coupling in various preclinical and clinical studies [19, 20, 21].

This study aims to demonstrate the feasibility of classifying myocardial infarct size, ischemic risk zone, and no‐reflow area in the acute MI setting using only carotid pressure waveform without requiring advanced imaging. Given that carotid pressure waveforms can be measured noninvasively, this proof‐of‐concept preclinical study holds translational potential for improving post‐MI patient management. It may enable timely interventions and enhanced monitoring to mitigate adverse outcomes.

## 2 Methods

### 2.1 Preclinical Data

In this study, we used a total number of *N* = 88 healthy Sprague‐Dawley female rats (∼200 g body weight; age of 7 ± 1 weeks [mean ± standard deviation]). The procedures were conducted based on the Guide for the Care and Use of Laboratory Animals (National Institutes of Health publication number 85‐23, National Academy Press, Washington, DC, revised 2011). All the procedures for the acute MI model (see “Standard rat model for acute myocardial infarction”) were approved by the Institutional Animal Care and Use Committee (IACUC) at the Huntington Medical Research Institutes (HMRI). These rats were part of other MI studies in our group, all of which followed the same myocardial infarction procedure, including: (1) the mitochondria‐targeted cytoprotective peptide SBT‐20 cardioprotective MI study [27] (control group i.e., no drug); (2) acute MI diagnostics via intrinsic frequencies study [21]; and (3) the smoking‐acute MI study [28] (control group i.e., air). Here, we used intrinsic frequency metrics of carotid pressure waveforms measured at 2 h after reperfusion.

### 2.2 Standard Rat Model for Acute Myocardial Infarction

We utilized the standard 30‐min coronary occlusion/3‐h reperfusion rat model in this study [29, 30], which is widely accepted for replicating key physiological aspects of human acute myocardial infarction, including myocardial infarct size, ischemic risk zone, and no‐reflow area. This model consistently produces a physiologically relevant and scalable range of injury sizes suitable for classification studies. The rats were anesthetized with intraperitoneal ketamine (75 mg/kg) and xylazine (5 mg/kg). Anesthesia depth was monitored by respiration, heart rate, toe pinch reflexes, and eye responses, with adjustments made as needed for movement or discomfort. Once stable anesthesia was confirmed, rats were intubated and ventilated at 60 breaths per minute, with a tidal volume of 1 mL/100 g body weight. The neck and chest were shaved and cleaned. A Millar catheter was inserted into the carotid artery to measure pressure, while a syringe was placed in the jugular vein for solution administration. An incision was made in the fourth left intercostal space to expose the heart. After removing the pericardium, a 4‐0 silk suture was placed under the left coronary artery, threaded through a tube, and clamped to induce coronary occlusion for 30 min. This is followed by coronary artery reperfusion via unclamping the coronary artery for 3 h. The carotid pressure waveforms, ECG signal, and temperature were continuously recorded throughout the surgeries. At the conclusion of 3‐h reperfusion, hearts were excised for triphenyl‐tetrazolium chloride (TTC) staining to quantify infarct size. TTC demarcates viable (red) versus infarcted (pale) myocardium, and is a validated histochemical gold standard for assessing myocardial necrosis [29]. Prior studies in rats have shown that TTC reliably stabilizes infarct boundaries after 1–2 h of reperfusion, and no further infarct growth is seen beyond this point [29]. For pressure waveform analysis, we selected the 2‐h post‐reperfusion timepoint for IF feature extraction. By this time, animals were reliably stable, whereas signal quality sometimes declined closer to the 3‐h endpoint (Supporting Information A). The body temperature was maintained constant at 37°C.

### 2.3 Measurement of Myocardial Infarct Size, Ischemic Risk Zone, and No‐Reflow Area

Before ending the surgeries, Thioflavin S solution was injected (while the epicardial coronary artery was patent) into the jugular vein to assess the no‐reflow area. In order to assess the ischemic risk zone, the left coronary artery was then re‐occluded, and Unisperse blue dye (Ciba‐Geigy, Hawthorne, NY, USA) was injected to mark perfused regions, leaving ischemic risk zones pink [29]. Potassium chloride (KCl) was then used to stop the heart at the end of the surgeries. The heart was excised and cleaned within saline. After the removal of the right ventricle and the major vessels, the left ventricle was transversely sliced into four sections and was prepared for photography.

**FIGURE 1:**
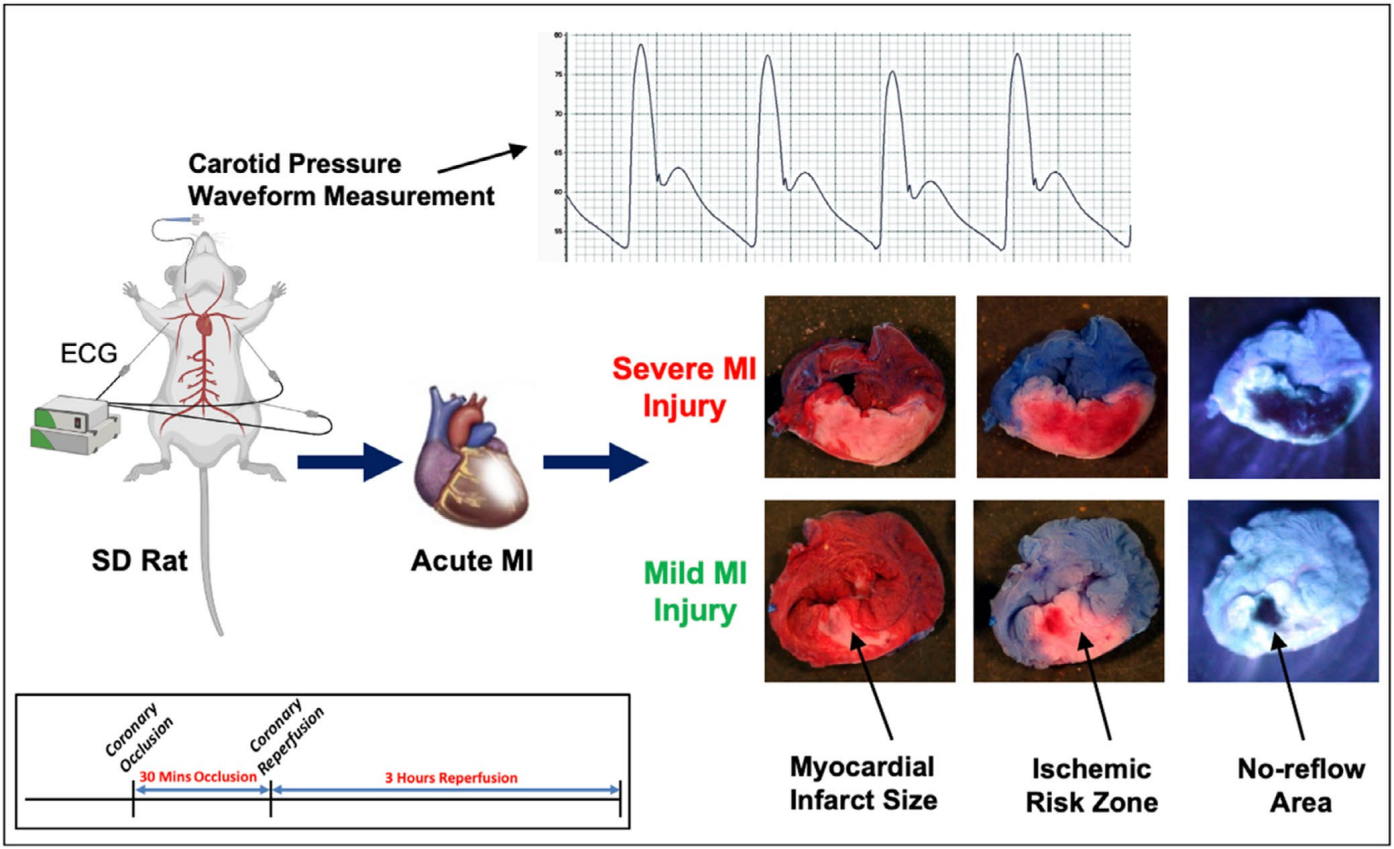
Overview of experimental workflow and myocardial injury sizes. (Top) Representative carotid pressure waveform recording in a Sprague‐Dawley (SD) rat undergoing acute myocardial infarction (MI) surgery. (Bottom left) Timeline of standard coronary occlusion (30 min)/reperfusion (3 h) model. (Right) Representative LV slices from two rats (one with mild and one with severe MI injury) showing myocardial infarct size (pale white tissues under white light), ischemic risk zone (pink tissues under white light), and no‐reflow area (dark or non‐fluorescent tissues under UV light).

The ischemic risk zone (pink area) and nonischemic zone (blue area) were photographed under white light (Figure 1), and the no‐reflow area (dark or non‐fluorescent area) and the areas of perfusion by Thioflavin S (light or fluorescent areas) were photographed under ultraviolet (UV) light (254 nm wavelength) (Figure 1). To measure myocardial infarct size, heart slices were then immersed in a 1% solution of TTC (a well‐established solution for heart and brain infarction visualization [21, 31, 32]) for 15 min at 37°C. The necrotic myocardial cells (white to yellow) and viable cells (stained, brick red) were then photographed (Figure 1). Using Image J software (National Institutes of Health, Bethesda, MD, USA), all the photographs were digitized for areas of infarction, ischemia, and no‐reflow, as well as total LV area. The corresponding mass of these areas was then calculated from the weight of each slice [29, 30]. Finally, all the MI injury sizes were calculated as mass percentages of LV or LV's ischemic zone.

The MI injury sizes that we measured in this study include myocardial infarct size, ischemic risk zone, and no‐reflow area. Myocardial infarct size, a measure of necrotic cells within the left ventricle (AN/LV), directly correlates with mortality and outcomes due to the myocardium's lack of regenerative capacity [33]. The ischemic risk zone of LV (AR/LV) represents the myocardial territory at risk during ischemia and influences the injury extent [34]. The no‐reflow area of LV (A‐NR/LV) denotes the regions where blood flow is not restored post‐reperfusion, correlating with microvascular damage, recovery, and outcomes [7]. Additionally, the myocardial infarct size (necrosis size) normalized by ischemic risk zone (AN/AR) and the no‐reflow area normalized by ischemic risk zone (A‐NR/AR) reflect the patient's resistance to acute MI. Smaller ratios indicate better resistance and recovery, while larger ratios suggest poorer recovery and higher mortality risk.

The distribution of MI injury sizes in our study was calculated as myocardial infarct size (AN/LV = 29.0% ± 11.4% [mean ± standard deviation (SD)], AN/AR = 58.2% ± 19.1%); ischemic risk zone (AR/LV = 49.5% ± 8.4%); and no‐reflow area (A‐NR/LV = 14.1% ± 7.8%, A‐NR/AR = 28.4% ± 14.3%). Representative transverse LV slice photographs demonstrating myocardial infarct size, ischemic risk zone, and no‐reflow area, along with the experimental design and timeline, are shown in Figure 1.

### 2.4 Invasive Hemodynamic Measurements

Throughout the surgical procedures, starting from post‐anesthesia and continued until 3 h post‐reperfusion, general tracings and hemodynamic parameters were continuously measured, monitored, and recorded. These included carotid pressure, electrocardiogram (ECG) signals (obtained via 29 gauge needle electrodes comprising three leads), as well as temperature readings. Carotid pressure waveforms were measured using a 2F high‐fidelity piezo‐tipped micro‐manometer (model SPR‐869, Millar Mikro‐Cath Pressure Catheter). Prior to each surgical session, thorough calibration of the catheter was performed. The body temperature was gauged and tracked utilizing a rectal thermocouple probe. The data acquisition and measurement process were facilitated by the LabChart‐Pro software (ADInstruments Ltd), operating through the PowerLab 4/35 data acquisition system (ADInstruments Ltd).

### 2.5 Cardiovascular Intrinsic Frequency Method

**FIGURE 2:**
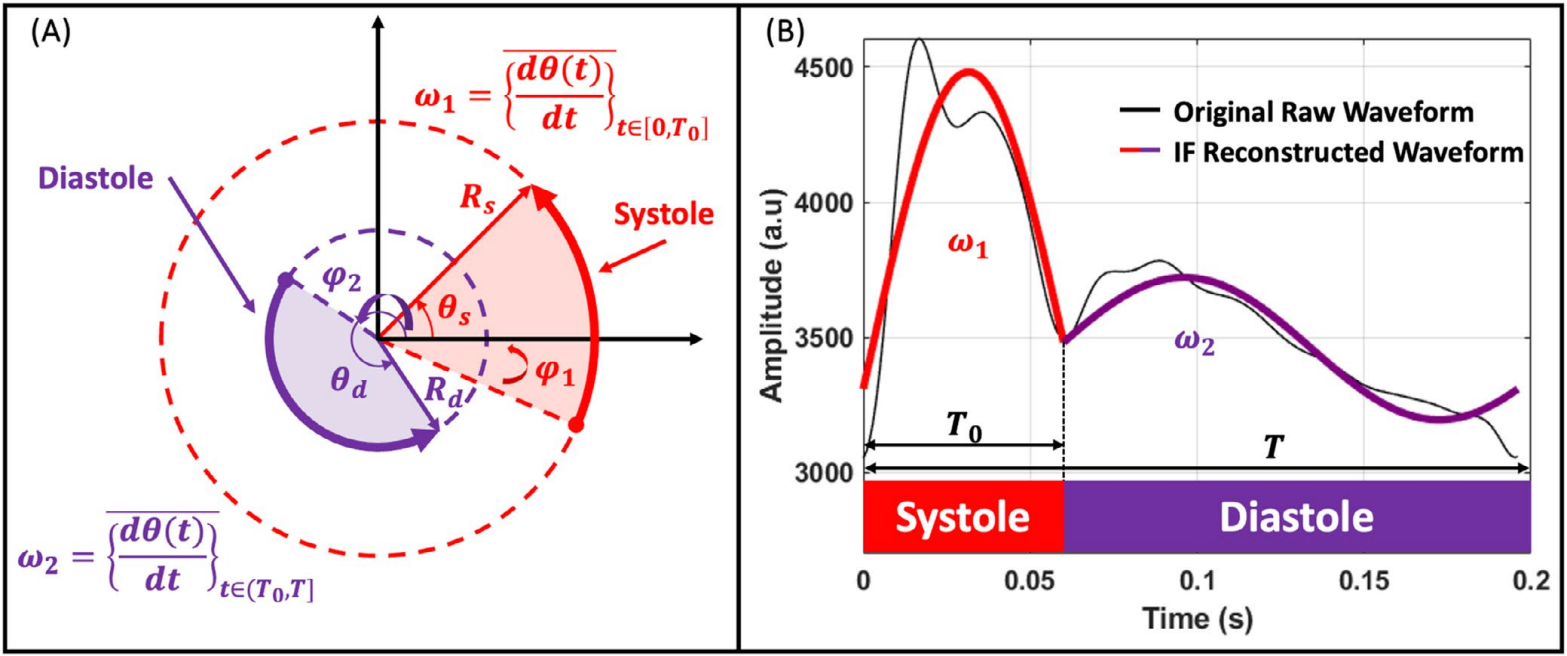
Visualization of the intrinsic frequency method. (A) Illustration of intrinsic frequency parameters across a full cardiac cycle. The IF metrics *ω*_1_ and *ω*_2_ represent the dominant operating frequencies (first and second intrinsic frequencies) during systole and diastole, respectively. The instantaneous frequency, *dθ*/*dt* captures dynamic frequency variations. The parameters *R*_s_ and *R*_d_ are the envelopes associated with *ω*_1_ and *ω*_2_, respectively, where *R*_s_ ≠ *R*_d_ in general. Additionally, *φ*_1_ and *φ*_2_ denote the initial intrinsic phases corresponding to *ω*_1_ and *ω*_2_, respectively. (B) A reconstructed carotid waveform based on intrinsic frequencies (depicted in red and purple) is overlaid on the original raw waveform (shown in black). The overlay, presented in arbitrary units (a.u), highlights the method's capability to reconstruct and analyze waveform dynamics.

The intrinsic frequency (IF) method analyzes arterial waveforms using a systems‐based sparse time‐frequency representation [19, 20, 21, 22, 24, 35, 36, 37]. It models the cardiovascular dynamics across systole and diastole (Figure 2A) via two dominant operating frequencies *ω*_1_ and *ω*_2_, obtained by minimizing the following objective function:

(1) $${\left\|p\left(t\right)-\chi (0,T_{0})\left[a_{1}\;\cos\left(\omega_{1}t\right)+b_{1}\;\sin\left(\omega_{1}t\;\right)\right]-\chi (T_{0},T)\left[a_{2}\;\cos\left(\omega_{2}t\right)+b_{2}\sin\;\left(\omega_{2}t\right)\right]-c\right\|}_{2}^{2}$$

where *ω*_1_ corresponds to systole (coupled LV‐Aortic dynamic system) and *ω*_2_ corresponds to diastole (decoupled arterial dynamics from LV). This minimization is subject to two nonlinear constraints: continuity at the decoupling time *t* = *T*_0_ (dicrotic notch; *T*_0_: systolic period) and waveform periodicity.

(2) $$\left\{\begin{array}{c} a_{1}\;\cos\left(\omega_{1}T_{0}\right)+b_{1}\;\sin\;\left(\omega_{1}T_{0}\right)=a_{2}\;\cos\left(\omega_{2}T_{0}\right)+b_{2}\sin\;\left(\omega_{2}T_{0}\right)\\ a_{1}=a_{2}\;\cos\left(\omega_{2}T\right)+b_{2}\sin\;\left(\omega_{2}T\right) \end{array}\right.$$

Here, *χ* (*α*, *β*) serves as an indicator function [*χ* (*α*, *β*) = 1 if *α* ≤ *t* ≤ *β* and *χ* (*α*, *β*) = 0 otherwise], *T*_0_ marks the end‐systolic time, *p*(*t*) is the input pressure waveform, and *T* is the cardiac cycle duration. Solving this minimization problem yields the intrinsic frequencies (*ω*_1_, *ω*_2_), fitting parameters (*a*_1_, *b*_1_, *a*_2_, *b*_2_), and a translation constant (*c*). Figure 2B shows a reconstruction of an arterial pressure waveform (a carotid waveform here) using the IF method. The total frequency variation (Δ*ω* = *ω*_1_ − *ω*_2_) has been proposed as a novel measure of optimal ventricular‐vascular coupling [19, 35]. *ω*_1_ and *ω*_2_ normalized via different approaches (as introduced by Tavallali et al. [38]) were also utilized (i.e., *ω*_1_ *T*_0_, *ω*_1_ *T*, *ω*_2_(*T* − *T*_0_), $\omega_{1}\sqrt{T_{0}}$). Additional IF metrics, defined using the trigonometric circle concept, include the intrinsic envelopes (i.e., systolic [*R*_s_] and diastolic [*R*_d_]), the envelope ratio (ER), and initial intrinsic phases (*φ*_1_ and *φ*_2_) (Figure 2A). For further details on the intrinsic frequency parameters and their physiological relevancy, see Table 1.

(3) $$R_{s}=\sqrt{a_{1}^{2}+b_{1}^{2}},R_{d}=\sqrt{a_{2}^{2}+b_{2}^{2}},\mathrm{ER}=\frac{R_{s}}{R_{d}}$$

(4) $$\varphi_{1}=\tan^{-1}\left(a_{1}/b_{1}\right),\varphi_{2}=\tan^{-1}\left(a_{2}/b_{2}\right)$$

**TABLE 1:** Physiological relevancy of the intrinsic frequency parameters.

IF parameter	Physiological relevancy	References
$\omega_{1},\varphi_{1},R_{s}$	LV contractile function and dynamics of coupled LV‐arterial system	[20, 21, 24, 39]
$\omega_{2},\varphi_{2},R_{d}$	Arterial dynamics and vascular function	[24, 25, 38]
Envelope ratio $\left(R_{s}/R_{d}\right)$	Combined influence of both LV function and vascular dynamics (LV‐arterial coupling)	[21, 24, 35]

In this study, we used a custom MATLAB script for solving the L2 optimization problem of the IF method [24], which requires approximately 3–5 s per waveform, depending on the optimization settings, when run on a laptop with a 2.3 GHz Quad‐Core Intel Core i5 processor [40].

### 2.6 Physics‐Based Feature Selection for Machine Learning

One of the main steps for a successful training and development of ML algorithms, especially when limited data is available, is a careful selection of appropriate inputs based on the underlying physics and physiology of the problem. To this end, we utilized the relevant IF metrics based on previous clinical and preclinical studies on myocardial infarction and ischemia [21, 41, 42, 43, 44], as well as systolic and diastolic pressures of the carotid waveform (SBP, DBP), end systolic blood pressure (*P*_end systolic_), and diastolic peak (maximum) pressure (DPP) values. Searching across all the possible variations of these parameters, the best input sets were selected based on the training and validation performance of our ML models. All the above features were extracted from invasively measured carotid pressure waveforms at a well‐established MI timepoint (i.e., 2 h post reperfusion of the 30 min occlusion, acute MI timepoint). Further details regarding the procedure of our physics‐based hybrid IF‐ML approach and the flowchart diagram illustrating the entire workflow can be found in the Supporting Information C.

### 2.7 Machine Learning Procedure and Evaluation

We employed multiple classifier algorithms, including K‐Nearest Neighbor (KNN) and Support Vector Classifier (SVC), along with five additional ML algorithms (detailed in Supporting Information D). We implemented the *k*‐fold cross‐validation (CV) technique and a stratified blind test, also referred to as ‘external validation’, to robustly assess the classifiers' performance. For parameter selection, we conducted a comprehensive, physics‐based search over combinations of intrinsic frequency (IF) metrics and key hemodynamic parameters informed by cardiovascular physiology. This targeted approach enabled efficient identification of optimal input sets for each injury metric while reducing computational cost and data requirements. The parameter selection process was guided exclusively by SVC classifier performance. To ensure consistency across models, the parameter sets selected by the SVC were then fixed and applied to all other classifiers (i.e., KNN, MLP, RF, LR, AdaBoost, and NB) for comparative analysis. The model outputs were defined as binary classifications of the waveforms, where the classifications were structured as [mild; severe] for three critical MI‐injury sizes. Specific cutoff values were selected based on thresholds established in previous studies when available [45, 46, 47]; otherwise, the median of our dataset was used to ensure balanced data distribution across each class of the targeted parameters (see Figure 4; Supporting Information E). For infarct size (normalized by LV), 0%–30% was considered mild and above 30% was considered severe [45, 46, 47]. For infarct size (normalized by the risk zone), 0%–60% was classified as mild and above 60% as severe. For the risk zone (normalized by LV), 0%–50% was classified as mild and above 50% as severe. For the no‐reflow area (normalized by LV), 0%–15% was considered mild and above 15% as severe. For the no‐reflow area (normalized by the risk zone), 0%–30% was considered mild and above 30% as severe.

The hyper‐parameters that were optimized in the training/validation process for SVCs included the Kernel function (i.e., linear, polynomial [poly], radial basis function [rbf], sigmoid), degree (i.e., 2,3) and Kernel coefficient (i.e., scale, auto). For KNNs, the hyperparameters included the number of neighbors (*n* = 2–12). The training accuracy of our ML models was evaluated by multiple parameters such as training score, CV average score, specificity, sensitivity, overall accuracy, and the area under the curve (AUC) defined by receiver operating characteristic (ROC). Sensitivity, specificity, and accuracy are defined as:

$$\text{Sensitivity}=\left(\text{true positive}\right)/\left(\text{true positive}+\text{false negative}\right)$$

$$\text{Specificity}=\left(\text{true negative}\right)/\left(\text{true negative}+\text{false positive}\right)$$

$$\text{Accuracy}=\left(\text{true positive}+\text{true negative}\right)/\left(\mathrm{all}\;\text{positive}+\mathrm{all}\;\text{negative}\right)$$

### 2.8 Data Specifications for Training and Blind Test

In this study, the total number of *n* = 88 rats was used, so *n* = 88 carotid waveforms were included in our acute MI study (i.e., from 2 h post‐reperfusion for each rat). Prior to parameter selection and development of the ML models, 25% of the rats (*n* = 22) were put aside for the stratified blind test. The remaining 75% (*n* = 66 waveforms) were used for developing (i.e., training and validation) the IF‐ML models.

## 3 Results

**FIGURE 3:**
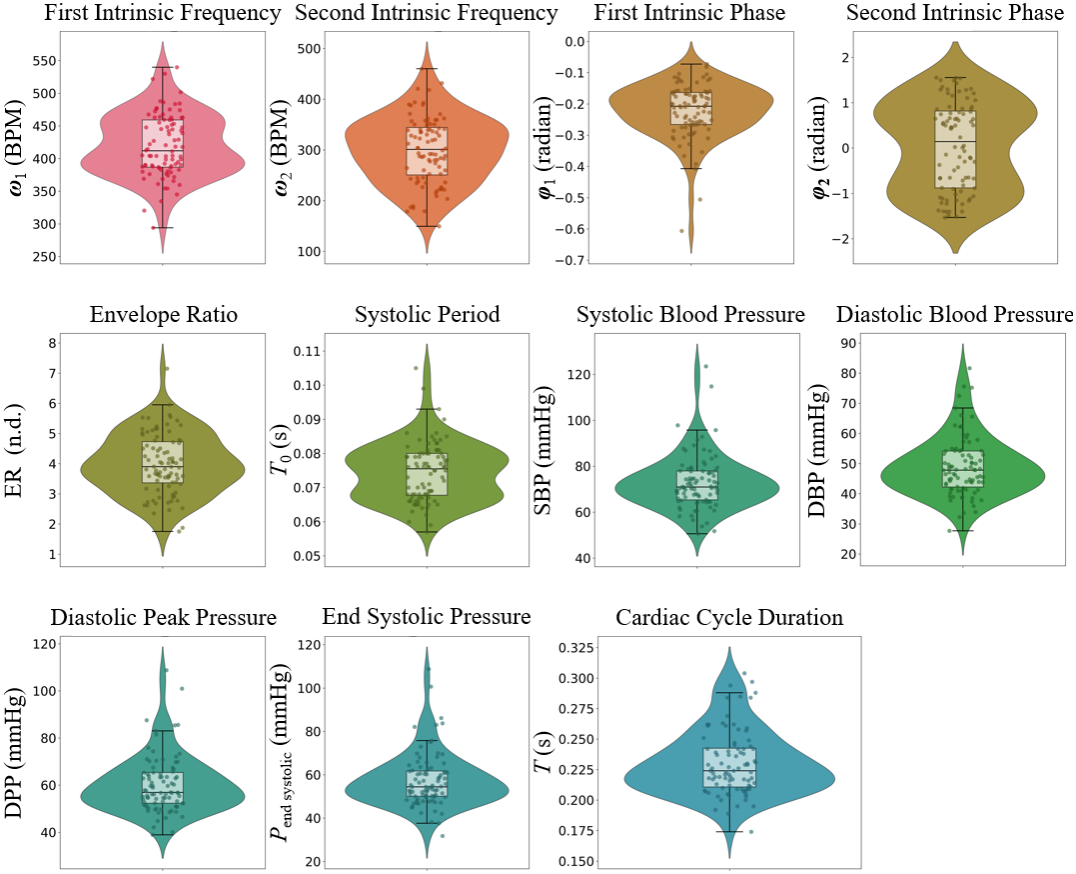
Violin plots with embedded box‐whisker plots of intrinsic frequency metrics and hemodynamic features across all 88 rats, showing distribution, median, and variability for each parameter.

**FIGURE 4:**
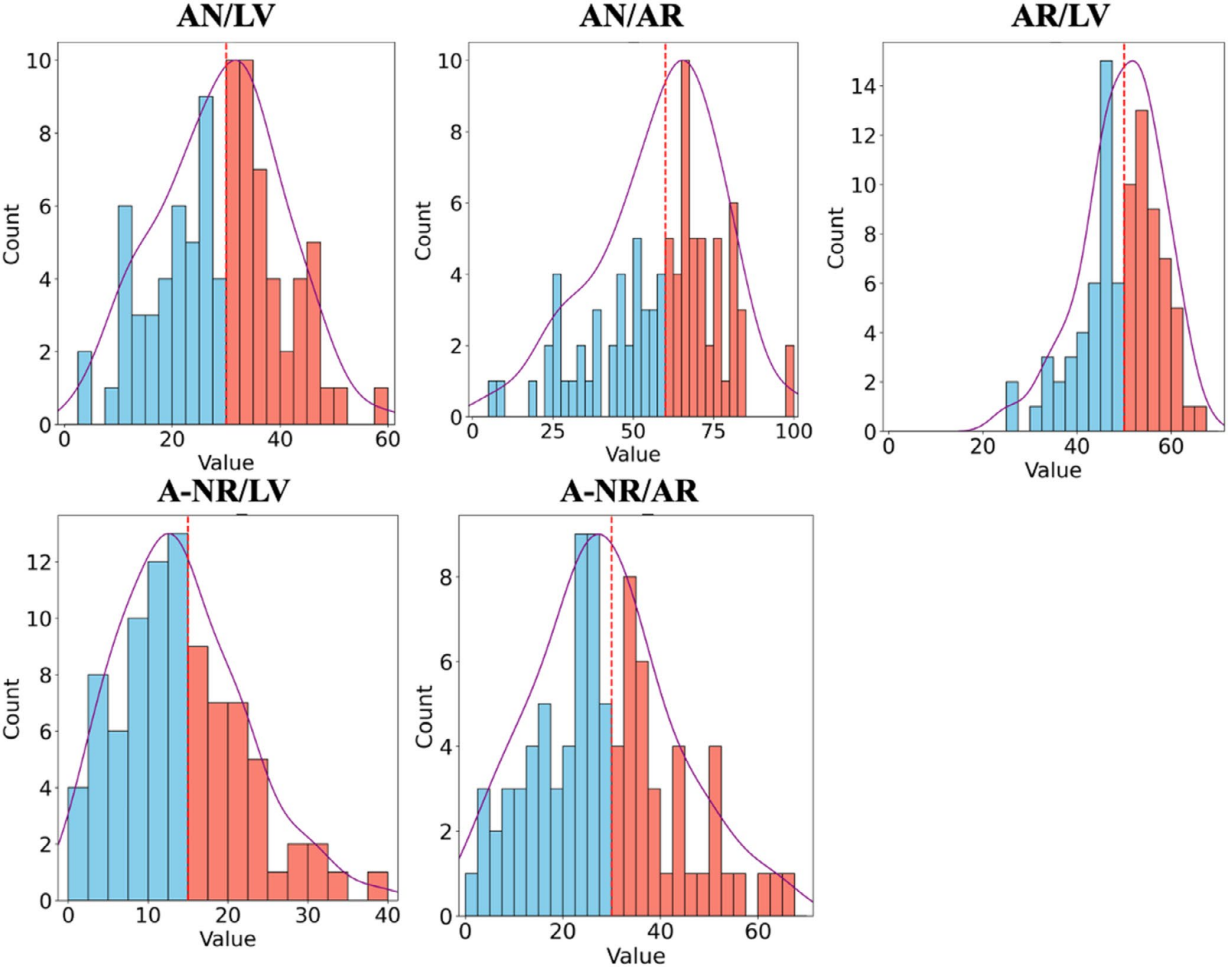
Histograms of myocardial infarction injury metrics for all rats. Blue and red bars represent mild and severe classes, respectively; red dashed lines indicate classification thresholds.

Table 2 presents baseline hemodynamics, IF metrics, and measurements of myocardial infarct size, ischemic risk zone, and no‐reflow area corresponding to all rats, the ML design rats (used for ML model training and generalization), and the blind test rats (which were kept blind to all the stages of the ML model development). More details about MI‐injury sizes data distribution can be found in Figure 4 and Figure S3. Using the thresholds (discussed above in Section 2.7), we distributed the MI‐injury sizes into balanced classes corresponding to each size. Table 3 presents the distribution of each class corresponding to the MI‐injury sizes in this study. To visually summarize statistics, Figure 3 presents violin plots (with embedded box‐whisker plots) of the IF metrics and hemodynamic variables across the full cohort (*n* = 88), illustrating the spread, central tendency, and variability of each parameter. Figure 4 displays histograms of all myocardial injury metrics, with blue and red bars denoting mild and severe cases, respectively, and vertical dashed lines indicating the thresholds used for classification. These visualizations provide a clearer picture of the parameter distributions and class separability used in the study's ML pipeline.

**TABLE 2:** Baseline hemodynamics, intrinsic frequency metrics, and MI measurement results for all rats, machine learning design rats, and blind test rats.

Parameter	All rats	ML design rats	Blind test rats
Number, *n* (%)	88 (100%)	66 (75%)	22 (25%)
*ω*_1_ (bpm)	293.9–539.9 (419.5 ± 47.6)	320.3–539.9 (419.7 ± 45.4)	293.9–530.0 (418.6 ± 54.8)
*ω*_2_ (bpm)	149.4–460.0 (299.8 ± 65.0)	149.4–460.0 (304.7 ± 62.4)	179.1–420.5 (284.9 ± 71.6)
*φ*_1_ (radian)	−0.61 to −0.07 (−0.22 ± 0.09)	−0.61 to −0.07 (−0.21 ± 0.09)	−0.41 to −0.12 (−0.25 ± 0.06)
*φ*_2_ (radian)	−1.53–1.55 (0.05 ± 0.97)	−1.53–1.53 (0.08 ± 0.97)	−1.48–1.55 (−0.06 ± 0.95)
ER	1.75–7.15 (3.98 ± 1.00)	1.75–7.15 (4.07 ± 1.03)	2.52–5.62 (3.73 ± 0.87)
*T*_0_ (s)	0.057–0.105 (0.070 ± 0.009)	0.057–0.099 (0.074 ± 0.008)	0.060–0.105 (0.070 ± 0.010)
DBP (mmHg)	27.7–81.6 (48.9 ± 9.9)	27.7–81.6 (48.4 ± 10.3)	32.3–72.5 (50.4 ± 8.7)
SBP (mmHg)	50.6–123.5 (72.9 ± 11.9)	50.6–123.5 (72.8 ± 12.0)	53.8–97.8 (73.1 ± 11.8)
DPP (mmHg)	38.9–108.8 (60.3 ± 12.2)	38.9–108.8 (59.8 ± 12.6)	40.1–85.6 (61.7 ± 10.8)
*P*_end systolic_ (mmHg)	31.7–108.8 (57.4 ± 12.6)	31.7–108.8 (56.9 ± 13.0)	37.6–83.7 (59.2 ± 11.1)
*T* (=60/HR) (s)	0.174–0.305 (0.227 ± 0.027)	0.189–0.305 (0.227 ± 0.025)	0.174–0.297 (0.228 ± 0.032)
AN/LV (%)	4.1–59.9 (29.0 ± 11.4)	4.4–59.9 (29.9 ± 11.3)	4.1–45.3 (26.2 ± 11.3)
AN/AR (%)	6.8–99.9 (58.2 ± 19.1)	7.8–99.9 (59.9 ± 19.1)	6.8–77.0 (53.0 ± 18.8)
AR/LV (%)	25.2–66.8 (49.5 ± 8.4)	25.2–62.3 (49.6 ± 8.0)	25.3–66.8 (49.3 ± 9.8)
A‐NR/LV (%)	0.8–38.8 (14.1 ± 7.8)	0.8–38.8 (14.4 ± 7.9)	1.5–30.2 (13.2 ± 7.7)
A‐NR/AR (%)	1.4–65.3 (28.4 ± 14.3)	1.4–65.3 (29.2 ± 14.6)	2.6–50.5 (26.7 ± 13.6)

*Note:* Values are presented in range (mean value ± SD).

Abbreviations: AN/AR, myocardial infarct (necrosis) size normalized by ischemic risk zone; AN/LV, myocardial infarct (necrosis) size normalized by left ventricle; A‐NR/AR, no‐reflow area normalized by ischemic risk zone; A‐NR/LV, no‐reflow area normalized by left ventricle; AR/LV, ischemic risk zone normalized by left ventricle; bpm, beats per minute; DBP, diastolic blood pressure; DPP, diastolic peak pressure (maximum pressure value during diastole); ER, envelope ratio; *P*_end systolic_, end systolic blood pressure value; SBP, systolic blood pressure; *T*, cardiac cycle duration; *T*_0_, systolic period; *φ*_1_, first initial intrinsic phase; *φ*_2_, second initial intrinsic phase; *ω*_1_, first intrinsic frequency; *ω*_2_, second intrinsic frequency.

**TABLE 3:** Data distribution [number (%)] for each class of MI‐injury sizes.

MI‐injury size	All rats, *n* = 88 (100%)		ML design rats, *n* = 66 (75%)		Blind test rats, *n* = 22 (25%)	
	Mild	Severe	Mild	Severe	Mild	Severe
	*n* (%)	*n* (%)	*n* (%)	*n* (%)	*n* (%)	*n* (%)
AN/LV	43 (48.9)	45 (51.1)	31 (47.0)	35 (53.0)	12 (54.5)	10 (45.5)
AN/AR	40 (45.5)	48 (54.5)	27 (40.9)	39 (59.1)	13 (59.1)	9 (40.9)
AR/LV	42 (47.7)	46 (52.3)	32 (48.5)	34 (51.5)	10 (45.5)	12 (54.5)
A‐NR/LV	53 (60.2)	35 (39.8)	39 (59.1)	27 (40.9)	15 (68.2)	7 (31.8)
A‐NR/AR	51 (58.0)	37 (42.0)	38 (57.6)	28 (42.4)	13 (59.1)	9 (40.9)

*Note:* For AN/LV, 0%–30% is mild and above 30% is severe; for AN/AR, 0%–60% is mild and above 60% is severe; for AR/LV, 0%–50% is mild and above 50% is severe; for A‐NR/LV, 0%–15% is mild and above 15% is severe; for A‐NR/AR, 0%–30% is mild and above 30% is severe.

### 3.1 Machine Learning‐Based Classification Models for Myocardial Infarct Size

Table 4 summarizes the final models and presents their input parameters as well as different accuracy metrics corresponding to such models. The optimal classifier models for myocardial infarct size (normalized by either LV [AN/LV] or ischemic risk zone [AN/AR]) were selected based on the accuracy threshold criteria [21]. For AN/LV classification, the KNN‐1 model shows specificity of 0.88 [95% CI: 0.75–0.96] and sensitivity of 0.89 [95% CI: 0.76–0.96] in all data. The SVC‐1 model shows specificity of 0.95 [95% CI: 0.84–0.99] and sensitivity of 0.96 [95% CI: 0.85–0.99]. For AN/AR classification, the KNN‐2 model shows specificity of 0.93 [95% CI: 0.80–0.98] and sensitivity of 0.81 [95% CI: 0.67–0.91] in all data. The SVC‐2 model shows specificity of 0.88 [95% CI: 0.73–0.96] and sensitivity of 1.00 [95% CI: 0.93–1.00].

**TABLE 4:** Characteristics and accuracy summary of the best optimal classifier models selected for myocardial infarct size.

Model	Input parameters	Training score	CV average score	Training		Blind test		All data	
				Spec	Sens	Spec	Sens	Spec	Sens
AN/LV (%)									
KNN‐1	*ω*_1_, *T*_0_, *T*, DBP, SBP, ER, DPP	0.91	0.76	0.91	0.90	0.80	0.83	0.88	0.89
SVC‐1	*ω*_1_, *T*_0_, *T*, DBP, SBP, ER, DPP	1.00	0.71	1.00	1.00	0.83	0.80	0.95	0.96
AN/AR (%)									
KNN‐2	*φ*_2_, *ω*_1_, *T*_0_, *T*, SBP, *P*_end systolic_	0.89	0.76	1.00	0.82	0.77	0.78	0.93	0.81
SVC‐2	*φ*_2_, *ω*_1_, *T*_0_, *T*, SBP, *P*_end systolic_	0.97	0.86	0.93	1.00	0.77	1.00	0.88	1.00

Abbreviations: KNN, K‐Nearest Neighbor classifier; Sens, sensitivity; Spec, specificity; SVC, Support Vector Classifier.

**FIGURE 5:**
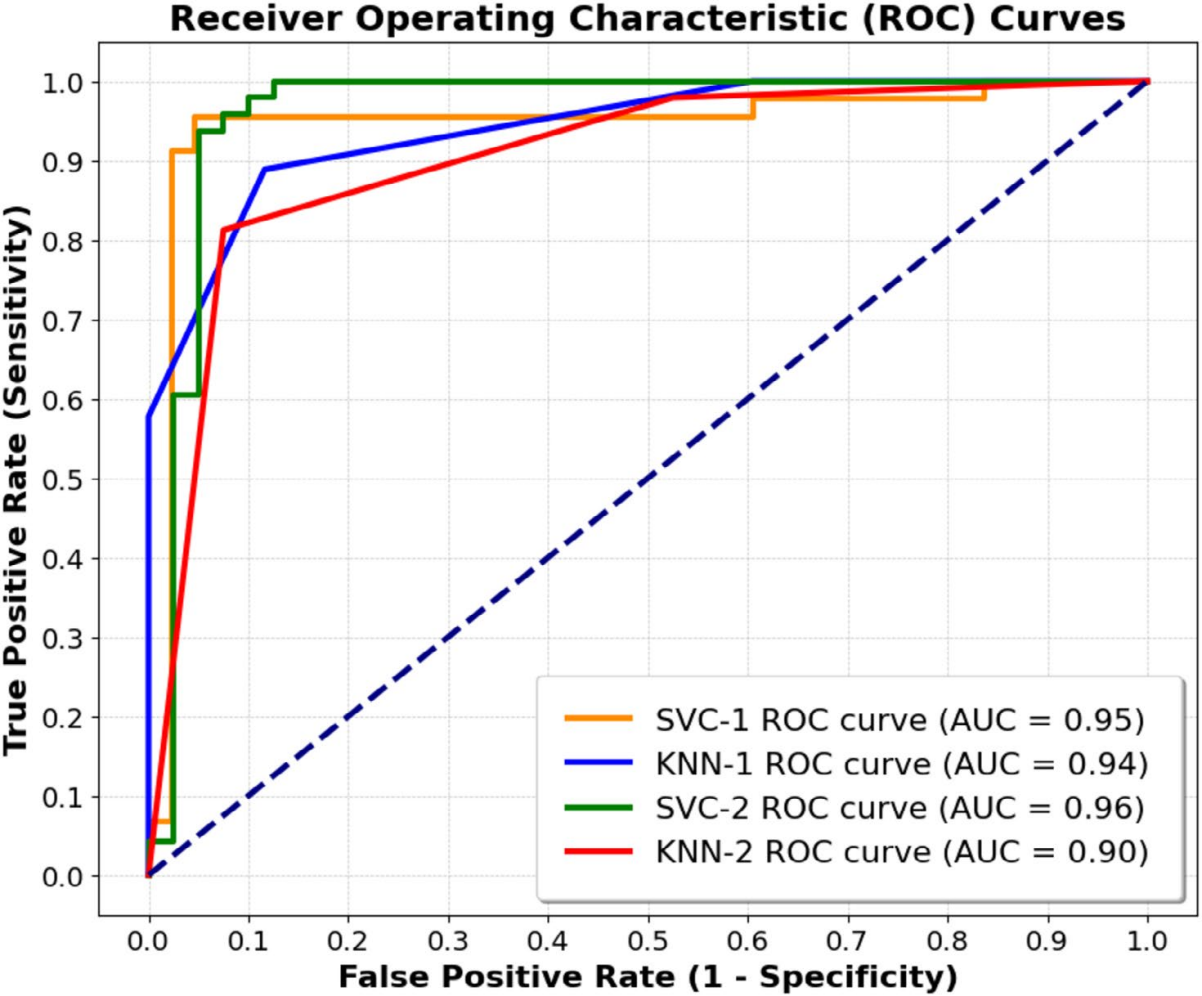
Receiver operating characteristic (ROC) curves of the selected models for classification of myocardial infarct size using all data. Area under curve (AUC) of each ROC curve is shown within the figure.

Figure 5 shows the receiver operating characteristics (ROC) curves of the selected models for classification of myocardial infarct size using all data. The AUC of each ROC is also computed and shown within Figure 5. Comprehensive details regarding the optimal hyperparameters for each classifier are provided in Supporting Information B, including a summary in Table S1. The confusion matrices for the selected models can be found in Supporting Information F and are visualized in Figure S4.

### 3.2 Machine Learning‐Based Classification Models for Ischemic Risk Zone

**FIGURE 6:**
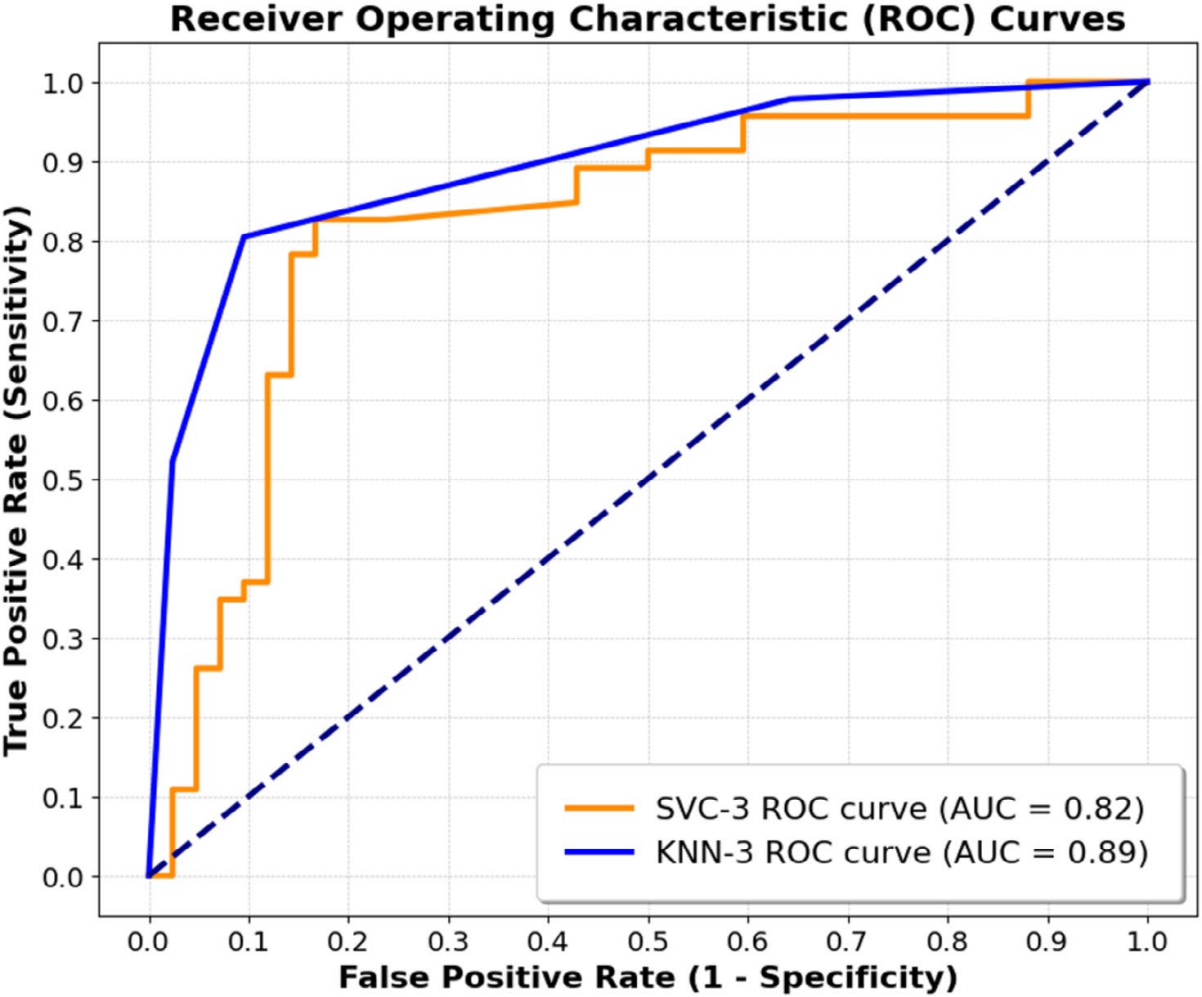
Receiver operating characteristic (ROC) curves of the selected models for classification of ischemic risk zone using all data. Area under curve (AUC) of each ROC curve is shown within the figure.

The optimal classifier models for ischemic risk zone (normalized by LV [AR/LV]) were picked based on our accuracy threshold criteria [21]. Table 5 summarizes the final models and presents their input parameters as well as different accuracy metrics corresponding to such models. For AR/LV classification, the KNN‐3 model shows specificity of 0.90 [95% CI: 0.77–0.97] and sensitivity of 0.80 [95% CI: 0.66–0.91] in all data. The SVC‐3 model shows specificity of 0.83 [95% CI: 0.69–0.93] and sensitivity of 0.78 [95% CI: 0.64–0.89]. Figure 6 shows ROC curves of the selected models for classification of ischemic risk zone using all data. The AUC of each ROC is also computed and shown within Figure 6. Comprehensive details regarding the optimal hyperparameters for each classifier are provided in Supporting Information B, including a summary in Table S1. The confusion matrices for the selected models can be found in Supporting Information F and are visualized in Figure S4.

**TABLE 5:** Characteristics and accuracy summary of the best optimal classifier models selected for ischemic risk zone.

Model	Input parameters	Training score	CV average score	Training		Blind test		All data	
				Spec	Sens	Spec	Sens	Spec	Sens
AR/LV (%)									
KNN‐3	ER, *ω*_1_, *T*_0_, T	0.86	0.74	0.91	0.82	0.90	0.75	0.90	0.80
SVC‐3	ER, *ω*_1_, *T*_0_, T	0.79	0.71	0.81	0.76	0.90	0.83	0.83	0.78

Abbreviations: KNN, K‐Nearest Neighbor classifier; Sens, sensitivity; Spec, specificity; SVC, Support Vector Classifier.

### 3.3 Machine Learning‐Based Classification Models for No‐Reflow Area

The optimal classifier models for no‐reflow area (normalized by either LV [A‐NR/LV] or ischemic risk zone [A‐NR/AR]) were picked based on our accuracy threshold criteria [21]. Table 6 summarizes the final models and presents their input parameters as well as different accuracy metrics corresponding to such models. For A‐NR/LV classification, the KNN‐4 model shows specificity of 0.83 [95% CI: 0.71–0.92] and sensitivity of 0.79 [95% CI: 0.62–0.91] in all data. The SVC‐4 model shows specificity of 0.87 [95% CI: 0.75–0.95] and sensitivity of 0.85 [95% CI: 0.69–0.95]. For A‐NR/AR classification, the KNN‐5 model shows specificity of 0.86 [95% CI: 0.74–0.94] and sensitivity of 0.84 [95% CI: 0.68–0.94] in all data. The SVC‐5 model shows specificity of 0.90 [95% CI: 0.79–0.97] and sensitivity of 0.81 [95% CI: 0.65–0.92].

**TABLE 6:** Characteristics and accuracy summary of the best optimal classifier models selected for no‐reflow area.

Model	Input parameters	Training score	CV average score	Training		Blind test		All data	
				Spec	Sens	Spec	Sens	Spec	Sens
A‐NR/LV (%)									
KNN‐4	*φ*_1_, ER, *ω*_1_, *T*_0_, *T*, DBP, DPP	0.86	0.76	0.89	0.82	0.73	0.71	0.83	0.79
SVC‐4	*φ*_1_, ER, *ω*_1_, *T*_0_, *T*, DBP, DPP	0.92	0.71	0.95	0.89	0.73	0.71	0.87	0.85
A‐NR/AR (%)									
KNN‐5	*φ*_1_, *ω*_1_, *T*_0_, *T*, SBP	0.85	0.73	0.84	0.86	0.92	0.78	0.86	0.84
SVC‐5	*φ*_1_, *ω*_1_, *T*_0_, *T*, SBP	0.94	0.74	1.00	0.86	0.62	0.67	0.90	0.81

Abbreviations: KNN, K‐Nearest Neighbor classifier; Sens, sensitivity; Spec, specificity; SVC, Support Vector Classifier.

**FIGURE 7:**
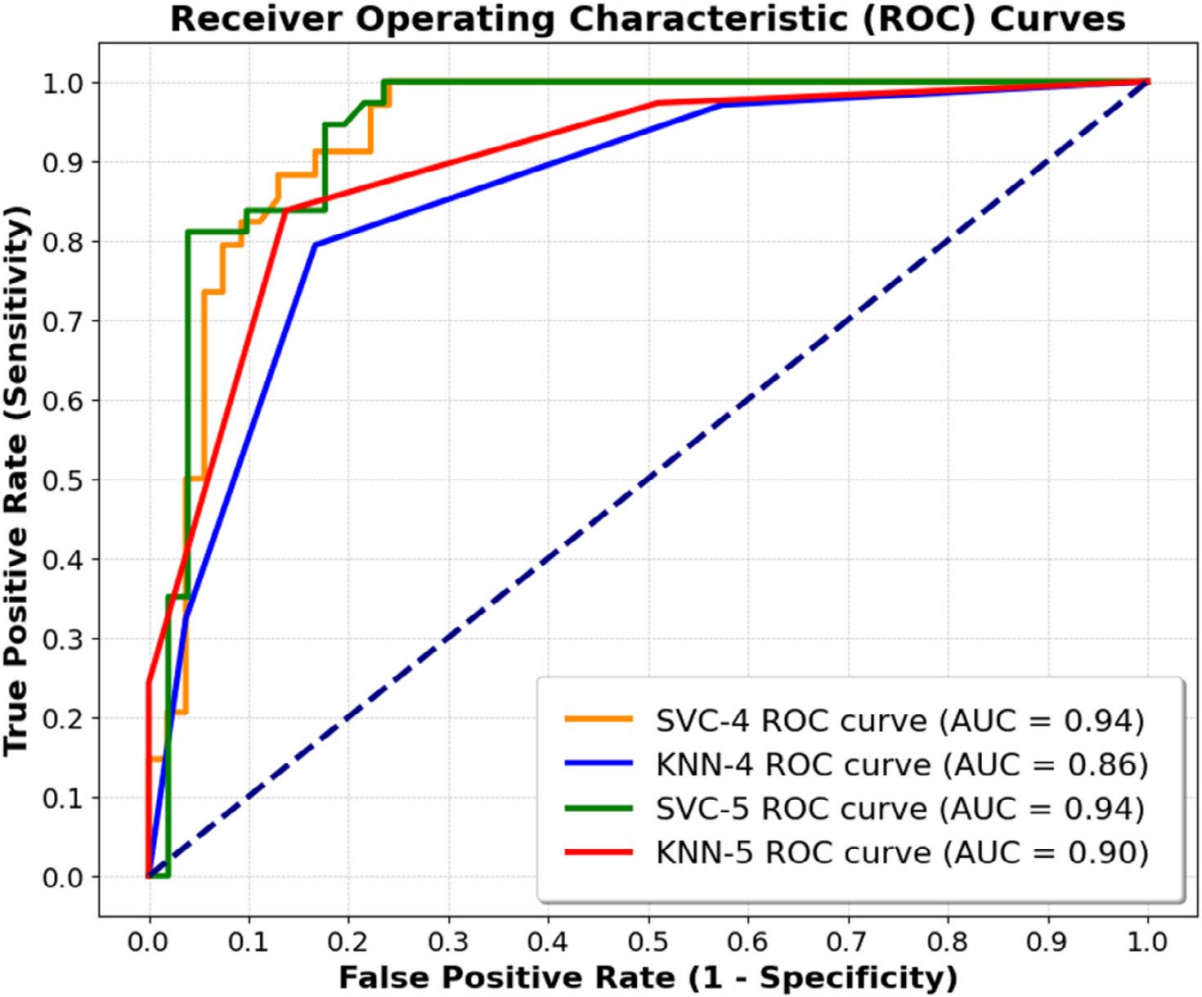
Receiver operating characteristic (ROC) curves of the selected models for classification of no‐reflow area using all data. Area under curve (AUC) of each ROC curve is shown within the figure.

Figure 7 shows the ROC curves of the selected models for classification of no‐reflow area using all data. The AUC of each ROC is also computed and shown within Figure 7. Comprehensive details regarding the optimal hyperparameters for each classifier are provided in Supporting Information B, including a summary in Table S1. The confusion matrices for the selected models can be found in Supporting Information F and are visualized in Figure S4.

## 4 Discussion

Conventional methods for determining MI injury sizes (i.e., myocardial infarct size, ischemic risk zone, and no‐reflow area) include MRI [14], CT [15], SPECT [16], and PET [17]. These methods require expert interpretation and have limitations such as time to analyze, radiation, high cost, and invasiveness. In this study, we used a standard occlusion/reperfusion rat model to demonstrate a preclinical proof‐of‐concept for a hybrid physics‐based machine learning approach that assesses the severity of various indicators of MI (MI‐injury sizes) from a single carotid pressure waveform. Our approach is totally independent of imaging techniques and shows promise for future clinical studies to assess MI severity.

ML methodologies are increasingly being used in medicine for prognostics, diagnosis, and patient management, particularly in cardiovascular diseases like hypertension, hypotension, and heart failure [48, 49]. Their robustness, accuracy, and universality make ML models powerful tools for early diagnosis and remote health monitoring. Physics‐informed ML models, which use systems‐level, reduced‐order, or non‐dimensionalized quantities, have shown promising results in assessing total arterial compliance, arterial pulse wave velocity, coronary artery disease risk, cardiac contractility, diastolic dysfunction, and aortic characteristic impedance [25, 38, 50, 51, 52, 53]. In this study, we successfully used the IF method and developed hybrid physics‐based ML models to assess the MI severity accurately.

During both the ischemic and infarction periods of MI progression, the cardiovascular system undergoes various regulatory responses that affect the hemodynamics of the arterial system and LV‐arterial coupling [21, 29]. Main changes due to MI include a decrease in LV's contractility, LV wall stiffening, and therefore an increase in LV end‐diastolic pressure (LVEDP), and a reduction in cardiac output [21]. Based on the hemodynamic dependency of the IF metrics (a physics‐informed approach) to the above‐mentioned changes (as established in previous preclinical and clinical studies [19, 20, 21, 24, 54, 55, 56]), we selected relevant inputs from different combinations of IF metrics into our ML algorithms (see Section 2 for details). Our proposed IF‐ML approach performed accurately for classifying all the MI‐injury sizes. However, the accuracy of the models developed for classifying the infarction size performed relatively better (e.g., perfect sensitivity by the model SVC‐2) compared to the ones developed for assessing the ischemic risk zone and no‐reflow area. This is more likely due to the fact that infarcted cells do not contribute to the LV contraction anymore, which directly impacts the arterial hemodynamics post‐MI (such as carotid pressure waveforms as an output of LV–arterial interactions). Therefore, carotid waveforms carry more information regarding the severity of infarction size compared to the risk zone and no‐reflow area. It should be noted that the ischemic risk zone creates high hemodynamic complexity due to including a mixed zone of myocardial cells in terms of functionality (i.e., a mixture of viable and infarcted cells), making its classification problem more difficult compared to other MI‐injury sizes. However, we have successfully developed classification models for the ischemic risk zone that could completely satisfy our accuracy threshold criteria.

Examining the final models, our results indicated that the first intrinsic frequency (*ω*_1_) was the primary determinant for classifying MI injury metrics, as it was consistently selected by all the ML models as a robust predictor (inputs). This finding is consistent with previous clinical studies, which have shown that *ω*_1_ is strongly influenced by LV contractile function [20, 24, 39]. The initial intrinsic phases (*φ*_1_ and *φ*_2_), which encode critical information about the pressure‐flow phase shift and vascular function, were selected by ML models for certain MI injury metrics. This finding aligns with the role of vascular function in regulating LV‐arterial coupling adaptation following MI and is consistent with observations from recent related MI studies [21, 24, 38, 57]. Additionally, the envelope ratio (ER), representing the ratio of energy stored in the systolic phase of the pressure waveform to that in the diastolic phase, emerged as a frequent input for our ML models. Notably, ER is predominantly influenced by vascular dynamics, such as pulse wave velocity, total arterial compliance, and peripheral resistance, as well as LV‐arterial coupling [24, 35, 38]. Overall, the significance of these IF parameters is consistent with the hemodynamic changes that occur during coronary occlusion and acute MI, highlighting their relevance in capturing MI‐related alterations. As expected, systolic and diastolic blood pressures (SBP and DBP), along with diastolic peak pressure (DPP), systolic period (*T*_0_), and cardiac cycle duration (*T*), emerged as meaningful contributors to MI injury classification in our ML models. While not sufficient alone, these classic hemodynamic parameters provided complementary information that enhanced model performance when added to physics‐based parameters (IF metrics).

One of the most critical complications following MI is heart failure [4]. Epidemiological studies reported that about 40% of MI patients develop new‐onset heart failure [12], and myocardial injury sizes have been shown to carry prognostic value in the development of such heart failures. Notably, in heart failure with preserved ejection fraction (HFpEF), limitations in LV functional reserve and the resulting hemodynamic derangements have been correlated with the severity of myocardial injury [58]. Emerging evidence suggests that myocardial infarct size, in particular, is a key determinant of long‐term cardiac remodeling and heart failure phenotype. In a preclinical rat study, the critical infarct size required to induce ventricular remodeling, cardiac dysfunction, and heart failure was identified as 36%, 38%, and 40%, respectively [11]. Clinical data from the STREAM trial further support this, showing that larger myocardial infarcts are strongly associated with cardiogenic shock and congestive heart failure [59]. These findings underscore the importance of early MI injury size assessment in guiding prognosis and tailoring therapeutic strategies for post‐MI heart failures. Our proposed IF‐ML approach, which utilizes carotid pressure waveforms and bypasses the need for imaging modalities, provides a rapid, inexpensive, and non‐invasive alternative for estimating myocardial injury sizes in MI patients. By capturing key hemodynamic signatures linked to myocardial injury sizes (such as impaired contractility and vascular dynamics [19, 21]), our method holds significant promise for early risk stratification, patient management, and longitudinal monitoring of MI patients.

While this study focused on invasive measurements in a rat model, the translational potential of the IF method has been supported by previous studies. In a recent study, invasive and non‐invasive IF parameters were shown to be almost equivalent [60]. Additionally, IF parameters have been shown to be scalable across species from rats or rabbits to humans [22]. These studies provide a strong physiological basis for future application of our IF‐based approach in humans using non‐invasive acquisition methods (e.g., applanation tonometry [19], optical tonometry [61], smartphone [20]). However, further validation in non‐invasive data from human cohorts is necessary for the particular application presented in this manuscript.

### 4.1 Strengths and Limitations

The standard 30‐min coronary occlusion/reperfusion rat model used in this study is a well‐established approach that closely replicates human pathophysiology and aligns with clinical scenarios (see Lindsey et al. [29]). Our proposed hybrid approach only requires pressure measurement without any need for advanced imaging and relies on the IF method to classify the severity of different MI‐injury sizes (i.e., myocardial infarct size, ischemic risk zone, and no‐reflow area) and does not rely on the ML technique that is adopted. To show it, we applied additional ML methods. Such methods (i.e., AdaBoost and Random Forest Classifier) were used with the same design steps, data specifications, and the trained models eventually presented similar accuracies as the main ML methods that we used in this study (i.e., KNN and SVC) (more details on additional ML methods and results can be found in Supporting Information D). Therefore, our proposed methodology works accurately independent of the ML approach. Moreover, previous studies have validated non‐invasive measurements against invasively measured arterial pressure waveforms and IF metrics [60, 62, 63, 64], so all the required inputs for our proposed IF‐ML models can be measured non‐invasively (e.g., using tonometry, optical tonometers, or a smartphone camera) [19, 20, 61]. The scalability of IF parameters between different mammals (e.g., rats and rabbits) and humans has also been shown [22]. Therefore, although this study is an invasive preclinical validation on rats, our approach can be translated to humans, offering non‐invasive and instantaneous applications in clinics or at home.

This proof‐of‐concept study also has limitations that should be considered. Female rats were exclusively used due to prior findings showing no gender‐based differences in the extent of myocardial injury [65], and male rats had higher mortality from reperfusion‐induced arrhythmias [66]. Typically, in the standard 30‐min coronary occlusion/reperfusion rat model, male rats experience more ventricular arrhythmias, leading to a higher mortality rate during the experiments compared to female rats. After initial validation of this hybrid IF‐ML methodology on young and healthy female rats, future studies will be designed to also incorporate male rats, older rats, and unhealthy rats such as rats with cardiovascular complications (e.g., spontaneously hypertensive rats).

### 4.2 Future Works

Building upon this proof‐of‐concept study, several directions will be pursued to enhance the generalizability, clinical relevance, and scalability of the proposed IF‐ML framework. First, we plan to increase the sample size to enable more detailed modeling of infarct severity through regression and multi‐class classification approaches. This proof‐of‐concept study provided important insight into which IF parameters are most strongly associated with injury severity. These findings will help guide the development of models that can predict infarct size, risk zone, and no‐reflow as continuous outcomes. Second, while this initial validation was conducted exclusively in young, healthy female rats, future studies will incorporate male subjects, aged animals, and disease models such as spontaneously hypertensive rats to better reflect diverse patient populations and comorbid conditions. Third, although the current study used invasively acquired carotid waveforms, future work will include validation using non‐invasive carotid pressure measurements obtained via applanation tonometry [19], optical tonometers [61], or smartphone‐based sensors [20], consistent with previous studies validating the intrinsic frequency method across measurement modalities and species [22]. Fourth, future studies will employ newly developed time‐frequency ML transfer functions [67, 68] to use peripheral arterial waveforms (e.g., radial or brachial pressure waveforms, which are more accessible for wearable monitoring) as inputs to the MI injury size models. Finally, future studies will incorporate parallel imaging techniques such as cardiac MRI to enable direct comparisons with IF‐derived predictions and further validate the clinical utility of the proposed approach.

While MI injury metrics are inherently continuous, a binary classification framework is adopted in this proof‐of‐concept study due to limited dataset size and the need to ensure model robustness. This approach enables the classification of MI injury severity. However, more detailed evaluation, particularly of the ischemic risk zone, may present greater complexity, given its heterogeneous composition (e.g., coexistence of infarcted and viable myocardial tissues), which can result in a complex relationship with left ventricular function. Future work will build on this foundation by incorporating larger datasets to enable multi‐class classification and regression modeling.

## 5 Conclusions

In this paper, we proposed a novel hybrid method for instantaneous, non‐invasive, and inexpensive assessment of MI‐injury sizes including myocardial infarct size, ischemic risk zone, and no‐reflow area, using a single arterial pressure waveform (here, carotid pressure waveform). We used invasively measured carotid pressure waveforms from the standard coronary occlusion/reperfusion rat model to develop, validate, and blindly test our methodology. Our approach integrates the intrinsic frequency method combined with physics‐based machine learning, with a streamlined training procedure. This study also provides the proof‐of‐concept that information about the severity of an acute MI can be extracted from carotid pressure waveforms (independent of traditional imaging or invasive procedures). In the final application, our proposed technique would require a small hand‐held arterial pressure waveform recorder (e.g., smartphone camera, tonometer‐type devices) or a wearable device, all of which could communicate with smartphone applications.

## Author Contributions

Jiajun Li, Rashid Alavi, and Wangde Dai performed the research and acquired the data; all authors conceived and designed the research; all authors analyzed and interpreted the data; all authors were involved in drafting and revising the manuscript.

## Disclosure

Niema M. Pahlevan holds equity in Avicena LLC and has a consulting agreement with Avicena LLC. The remaining authors have no disclosures to report.

## Conflicts of Interest

The authors declare no conflicts of interest.

## Supporting information


**Data S1:** fsb271029‐sup‐0001‐DataS1.docx.

## Data Availability

The data that support the findings of this study are available upon request from the corresponding author. The data are not publicly available due to privacy or ethical restrictions.
