# Supplementary material for: Assessment of Myocardial Injury Size Metrics Using Carotid Pressure Waveform: Proof‐of‐Concept in Coronary Occlusion/Reperfusion Rat Model
Source: FASEB J. 2025 Sep 6;39(17):e71029. doi: 10.1096/fj.202502111R (PMC12413657; doi:10.1096/fj.202502111R)
Supplement: Supplementary file 1 — Data S1: fsb271029‐sup‐0001‐DataS1.docx. [file FSB2-39-e71029-s001.docx]

**Supplemental Appendix**

**Manuscript Title:**

*“Assessment of Myocardial Injury Size Metrics Using Carotid Pressure Waveform: Proof-of-Concept in Coronary Occlusion/Reperfusion Rat Model”*

Contents

[Supplement A: Surgical Timeline for Standard MI Rat Model 2](#_Toc205924932)

[Supplement B: Details of Optimal Hyperparameters 3](#_Toc205924933)

[Supplement C: The Overall Procedure of the Physics-based Hybrid IF-ML Approach 4](#_Toc205924934)

[Supplement D: Independency of the Proposed Hybrid IF-ML Method from the ML Approaches 5](#_Toc205924935)

[**Machine Learning-Based Classification Models for Myocardial Infarct Size** 5](#_Toc205924936)

[**Machine Learning-Based Classification Models for Ischemic Risk Zone** 6](#_Toc205924937)

[**Machine Learning-Based Classification Models for No-reflow Area** 6](#_Toc205924938)

[Supplement E: Data Points Distribution for Design, Blind Test and All Data in Boxplot 7](#_Toc205924939)

[Supplement F: Confusion Matrices of the Selected Models for Classification of MI-injury sizes 8](#_Toc205924940)

Supplement A: Surgical Timeline for Standard MI Rat Model

The overall surgical timeline, including the durations of occlusion and reperfusion, is illustrated in Figure S1.


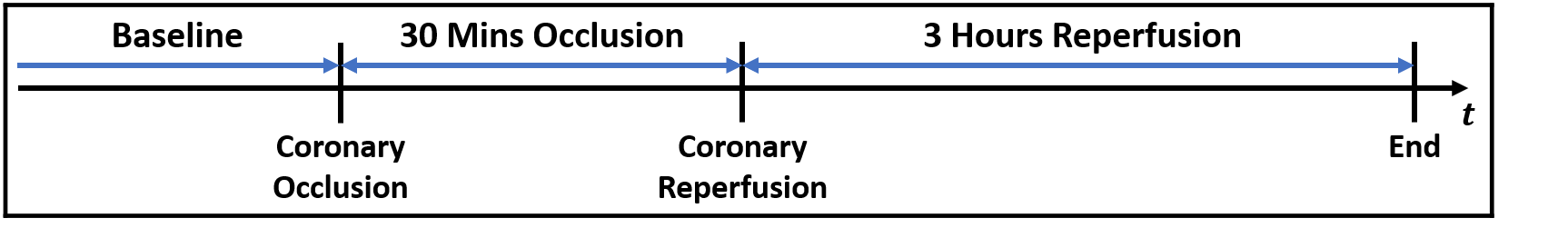


**Figure S1.** Surgical timeline for the standard myocardial infarction/ischemia rat model.

Supplement B: Details of Optimal Hyperparameters

**Table S1.** Optimal hyperparameters of the selected classification models designed for severity analysis of MI. k-fold cross-validation (k=5, random state=42) was performed during the model developments.

| Model | C | Kernel Function | Gamma (Kernel coefficient) | Tol (Tolerance) | Degree (Degree of the polynomial kernel funcion) | n_neighbors ( Number of neighbors) | algorithm | metric (distance function) |
| --- | --- | --- | --- | --- | --- | --- | --- | --- |
| **AN / LV** | | | | | | | | |
| KNN-1 | - | - | - | - | - | 3 | auto | chebyshev |
| SVC-1 | 24 | poly | 1.00 | 0.01 | 4 | - | - | - |
| **AN / AR** | | | | | | | | |
| KNN-2 | - | - | - | - | - | 2 | auto | chebyshev |
| SVC-2 | 10 | poly | 1 | 0.01 | 4 | - | - | - |
| **AR / LV** | | | | | | | | |
| KNN-3 | - | - | - | - | - | 3 | auto | correlation |
| SVC-3 | 31 | poly | scale | 0.01 | 2 | - | - | - |
| **A-NR / LV** | | | | | | | | |
| KNN-4 | - | - | - | - | - | 3 | auto | manhattan |
| SVC-4 | 15 | rbf | scale | 0.01 | 2 | - | - | - |
| **A-NR / AR** | | | | | | | | |
| KNN-5 | - | - | - | - | - | 3 | auto | correlation |
| SVC-5 | 40 | rbf | scale | 0.001 | 2 | - | - | - |

* C: Regularization parameter. The strength of the regularization is inversely proportional to C; AN/LV, myocardial infarct size normalized by left ventricle; AN/AR, myocardial infarct size normalized by ischemic risk zone; AR/LV, ischemic risk zone normalized by left ventricle; A-NR/LV, no-reflow area normalized by left ventricle; A-NR/AR, no-reflow area normalized by ischemic risk zone.

Supplement C: The Overall Procedure of the Physics-based Hybrid IF-ML Approach


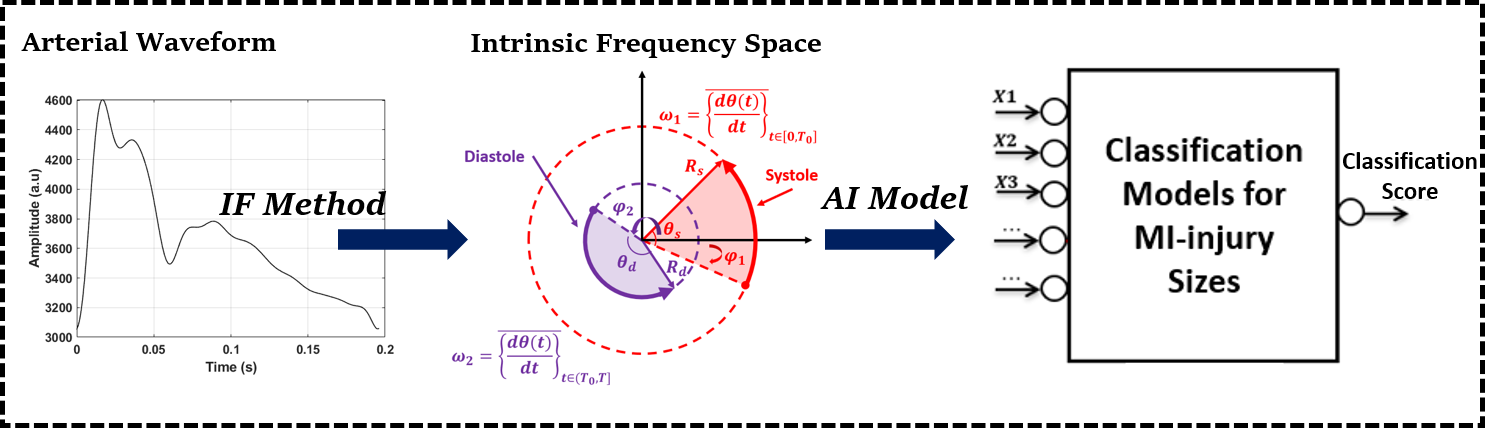


**Figure S2.** Flowchart of physics-based hybrid ML method for classifying MI-injury sizes. Physics-informed reduced-order parameters (X₁, X₂, …, Xₙ) are extracted and optimized for the best model design, integrating physiological insights with machine learning for accurate classification.

Supplement D: Independency of the Proposed Hybrid IF-ML Method from the ML Approaches

Five additional ML classifiers: Multilayer Perceptron (MLP), Random Forest (RF), Logistic Regression (LR), AdaBoost (Ada), and Naïve Bayes (NB), following the same design steps.

**Machine Learning-Based Classification Models for Myocardial Infarct Size**

**Table S2.** Characteristics and accuracy summary of the additional MLP, RF, LR, Ada, and NB models developed for myocardial infarct size normalized by both LV and ischemic risk zone.

| Model | Input parameters | Training score | CV average score | Training | | Blind test | | All data | |
| --- | --- | --- | --- | --- | --- | --- | --- | --- | --- |
|  |  |  |  | Spec | Sens | Spec | Sens | Spec | Sens |
| **AN/LV (%)** | | | | | | | | | |
| MLP-1 | ***ω***_1_, *T*_0_, *T*, *DBP*, *SBP*, ER, *DPP* | 1.00 | 0.74 | 1.00 | 1.00 | 0.75 | 0.90 | 0.93 | 0.98 |
| RF-1 | ***ω***_1_, *T*_0_, *T*, *DBP*, *SBP*, ER, *DPP* | 1.00 | 0.74 | 1.00 | 1.00 | 0.83 | 0.70 | 0.95 | 0.93 |
| LR-1 | ***ω***_1_, *T*_0_, *T*, *DBP*, *SBP*, ER, *DPP* | 0.76 | 0.71 | 0.71 | 0.80 | 0.75 | 0.70 | 0.72 | 0.78 |
| Ada-1 | ***ω***_1_, *T*_0_, *T*, *DBP*, *SBP*, ER, *DPP* | 1.00 | 0.73 | 1.00 | 1.00 | 0.92 | 0.80 | 0.98 | 0.96 |
| NB-1 | ***ω***_1_, *T*_0_, *T*, *DBP*, *SBP*, ER, *DPP* Diast_max_ | 0.74 | 0.73 | 0.77 | 0.71 | 0.75 | 0.70 | 0.77 | 0.71 |
| **AN/AR (%)** | | | | | | | | | |
| MLP-2 | ***φ***_2_, ***ω***_1_, *T*_0_, *T, SBP*, *P*_end systolic_ | 1.00 | 0.76 | 1.00 | 1.00 | 0.77 | 0.89 | 0.93 | 0.98 |
| RF-2 | ***φ***_2_, ***ω***_1_, *T*_0_, *T, SBP*, *P*_end systolic_ | 1.00 | 0.82 | 1.00 | 1.00 | 0.69 | 0.89 | 0.90 | 0.98 |
| LR-2 | ***φ***_2_, ***ω***_1_, *T*_0_, *T, SBP*, *P*_end systolic_ | 0.76 | 0.71 | 0.67 | 0.82 | 0.69 | 0.89 | 0.68 | 0.83 |
| Ada-2 | ***φ***_2_, ***ω***_1_, *T*_0_, *T, SBP*, *P*_end systolic_ | 1.00 | 0.76 | 1.00 | 1.00 | 0.69 | 0.89 | 0.90 | 0.98 |
| NB-2 | ***φ***_2_, ***ω***_1_, *T*_0_, *T, SBP*, *P*_end systolic_ | 0.79 | 0.79 | 0.67 | 0.87 | 0.62 | 0.89 | 0.65 | 0.88 |

* MLP, Multilayer Perceptron Classifier; RF, Random Forest Classifier; LR, Logistic Regression Classifier; Ada, AdaBoost Classifier; NB, Naïve Bayes Classifier; Spec, specificity; Sens, sensitivity

**Machine Learning-Based Classification Models for Ischemic Risk Zone**

**Table S3.** Characteristics and accuracy summary of the additional MLP, RF, LR, Ada, and NB models developed for ischemic risk zone normalized by LV.

| Model | Input parameters | Training score | CV average score | Training | | Blind test | | All data | |  |
| --- | --- | --- | --- | --- | --- | --- | --- | --- | --- | --- |
|  |  |  |  | Spec | Sens | Spec | Sens | Spec | Sens |  |
| **AR/LV (%)** | | | | | | | | | | |
| MLP-3 | ER, ***ω***_1_, *T*_0_, T | 0.80 | 0.71 | 0.81 | 0.79 | 0.90 | 0.83 | 0.83 | 0.80 |  |
| RF-3 | ER, ***ω***_1_, *T*_0_, T | 0.89 | 0.65 | 0.84 | 0.94 | 0.60 | 0.83 | 0.79 | 0.91 |  |
| LR-3 | ER, ***ω***_1_, *T*_0_, T | 0.61 | 0.60 | 0.56 | 0.65 | 0.90 | 0.75 | 0.64 | 0.67 |  |
| Ada-3 | ER, ***ω***_1_, *T*_0_, T | 0.67 | 0.59 | 0.75 | 0.59 | 0.75 | 0.73 | 0.74 | 0.63 |  |
| NB-3 | ER, ***ω***_1_, *T*_0_, T | 0.68 | 0.61 | 0.78 | 0.59 | 0.80 | 0.75 | 0.79 | 0.63 |  |

* MLP, Multilayer Perceptron Classifier; RF, Random Forest Classifier; LR, Logistic Regression Classifier; Ada, AdaBoost Classifier; NB, Naïve Bayes Classifier; Spec, specificity; Sens, sensitivity

**Machine Learning-Based Classification Models for No-reflow Area**

**Table S4.** Characteristics and accuracy summary of the additional MLP, RF, LR, Ada, and NB models developed for no-reflow area normalized by both LV and ischemic risk zone.

| Model | Input parameters | Training score | CV average score | Training | | Blind test | | All data | |
| --- | --- | --- | --- | --- | --- | --- | --- | --- | --- |
|  |  |  |  | Spec | Sens | Spec | Sens | Spec | Sens |
| **A-NR / LV (%)** | | | | | | | | | |
| MLP-4 | ***φ***_1_, ER, ***ω***_1_, *T*_0_, *T*, *DBP*, *DPP* | 1.00 | 0.73 | 1.00 | 1.00 | 0.80 | 0.71 | 0.93 | 0.94 |
| RF-4 | ***φ***_1_, ER, ***ω***_1_, *T*_0_, *T*, *DBP*, *DPP* | 0.89 | 0.79 | 0.92 | 0.86 | 0.80 | 0.57 | 0.87 | 0.79 |
| LR-4 | ***φ***_1_, ER, ***ω***_1_, *T*_0_, *T*, *DBP*, *DPP* | 0.80 | 0.72 | 0.84 | 0.75 | 0.87 | 0.43 | 0.83 | 0.68 |
| Ada-4 | ***φ***_1_, ER, ***ω***_1_, *T*_0_, *T*, *DBP*, *DPP* | 1.00 | 0.76 | 1.00 | 1.00 | 0.80 | 0.57 | 0.93 | 0.91 |
| NB-4 | ***φ***_1_, ER, ***ω***_1_, *T*_0_, *T*, *DBP*, *DPP* | 0.70 | 0.70 | 0.68 | 0.71 | 0.67 | 0.29 | 0.67 | 0.62 |
| **A-NR / AR (%)** | | | | | | | | | |
| MLP-5 | ***φ***_1_, ***ω***_1_, *T*_0_, *T*, *SBP* | 1.00 | 0.70 | 1.00 | 1.00 | 0.85 | 0.78 | 0.96 | 0.95 |
| RF-5 | ***φ***_1_, ***ω***_1_, *T*_0_, *T*, *SBP* | 1.00 | 0.76 | 1.00 | 1.00 | 0.92 | 0.22 | 0.98 | 0.81 |
| LR-5 | ***φ***_1_, ***ω***_1_, *T*_0_, *T*, *SBP* | 0.74 | 0.71 | 0.79 | 0.68 | 0.77 | 0.22 | 0.78 | 0.57 |
| Ada-5 | ***φ***_1_, ***ω***_1_, *T*_0_, *T*, *SBP* | 1.00 | 0.77 | 1.00 | 1.00 | 0.77 | 0.56 | 0.94 | 0.89 |
| NB-5 | ***φ***_1_, ***ω***_1_, *T*_0_, *T*, *SBP* | 0.77 | 0.70 | 0.79 | 0.75 | 0.69 | 0.33 | 0.76 | 0.65 |

* MLP, Multilayer Perceptron Classifier; RF, Random Forest Classifier; LR, Logistic Regression Classifier; Ada, AdaBoost Classifier; NB, Naïve Bayes Classifier; Spec, specificity; Sens, sensitivity

Supplement E: Data Points Distribution for Design, Blind Test and All Data in Boxplot

**
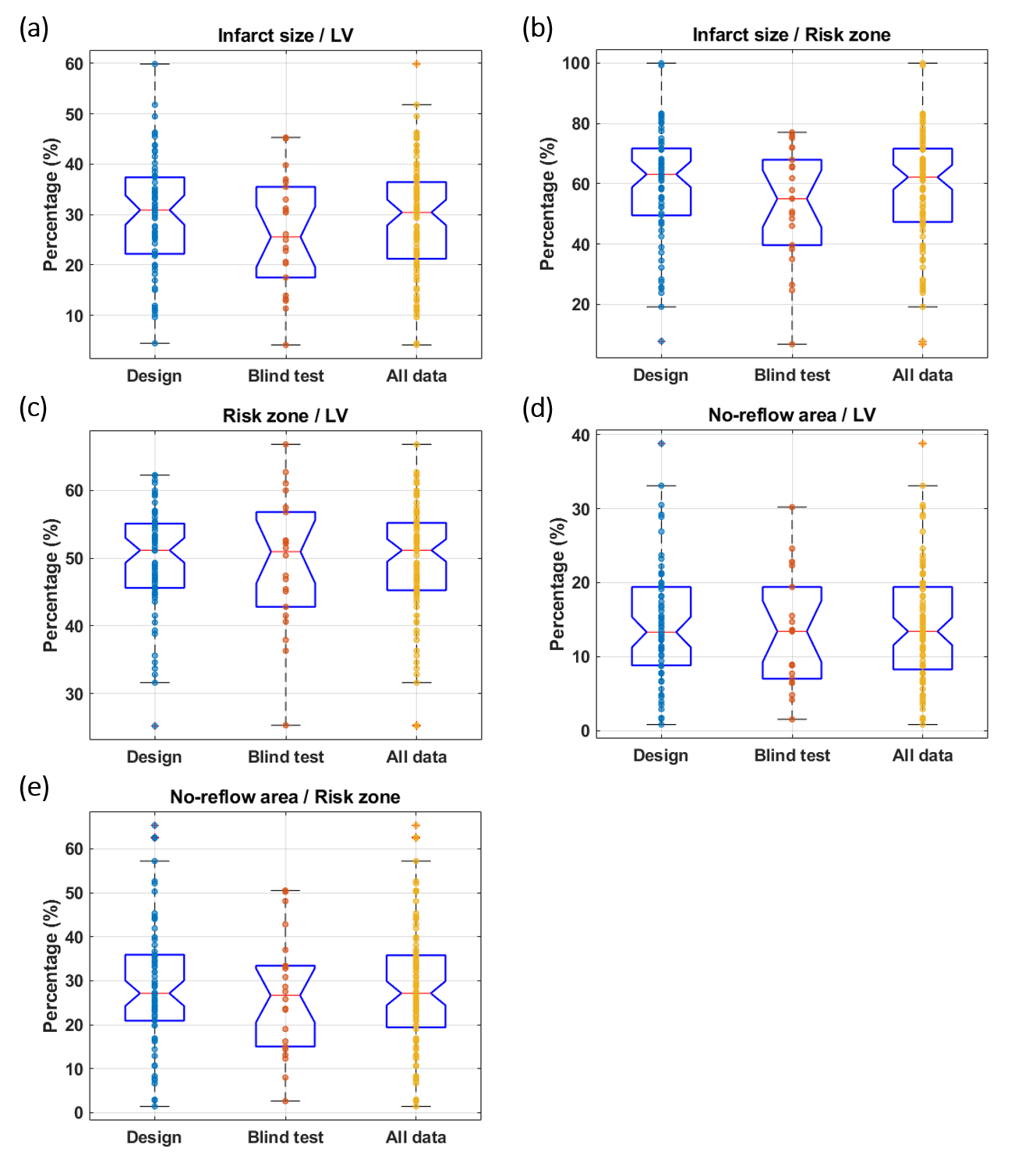
**

**Figure S3.** Data points distribution for design, blind test and all data in boxplot for (a) AN/LV, (b) AN/AR, (c) AR/LV, (d) A-NR/LV, (e) A-NR/AR.

Supplement F: Confusion Matrices of the Selected Models for Classification of MI-injury sizes


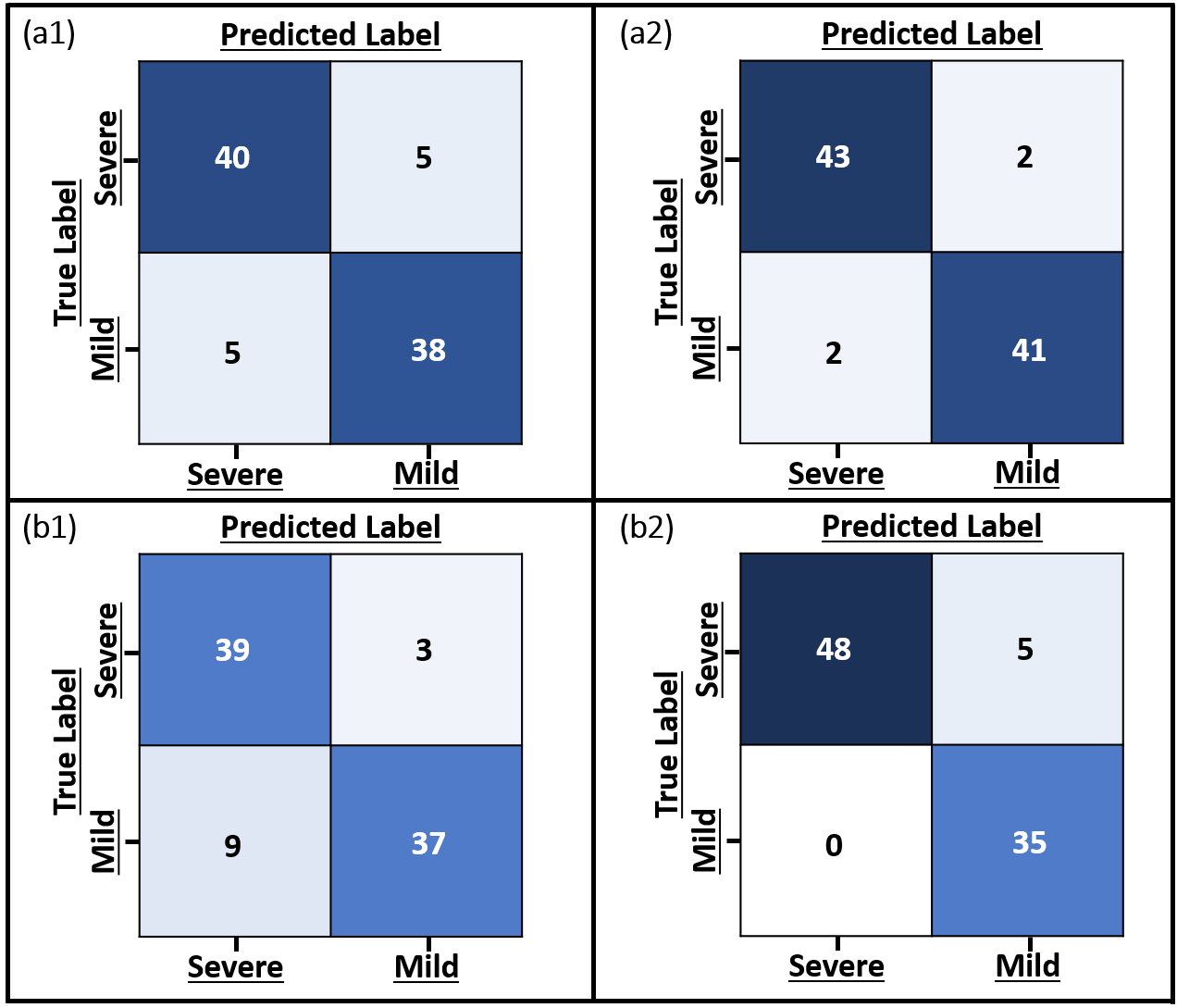


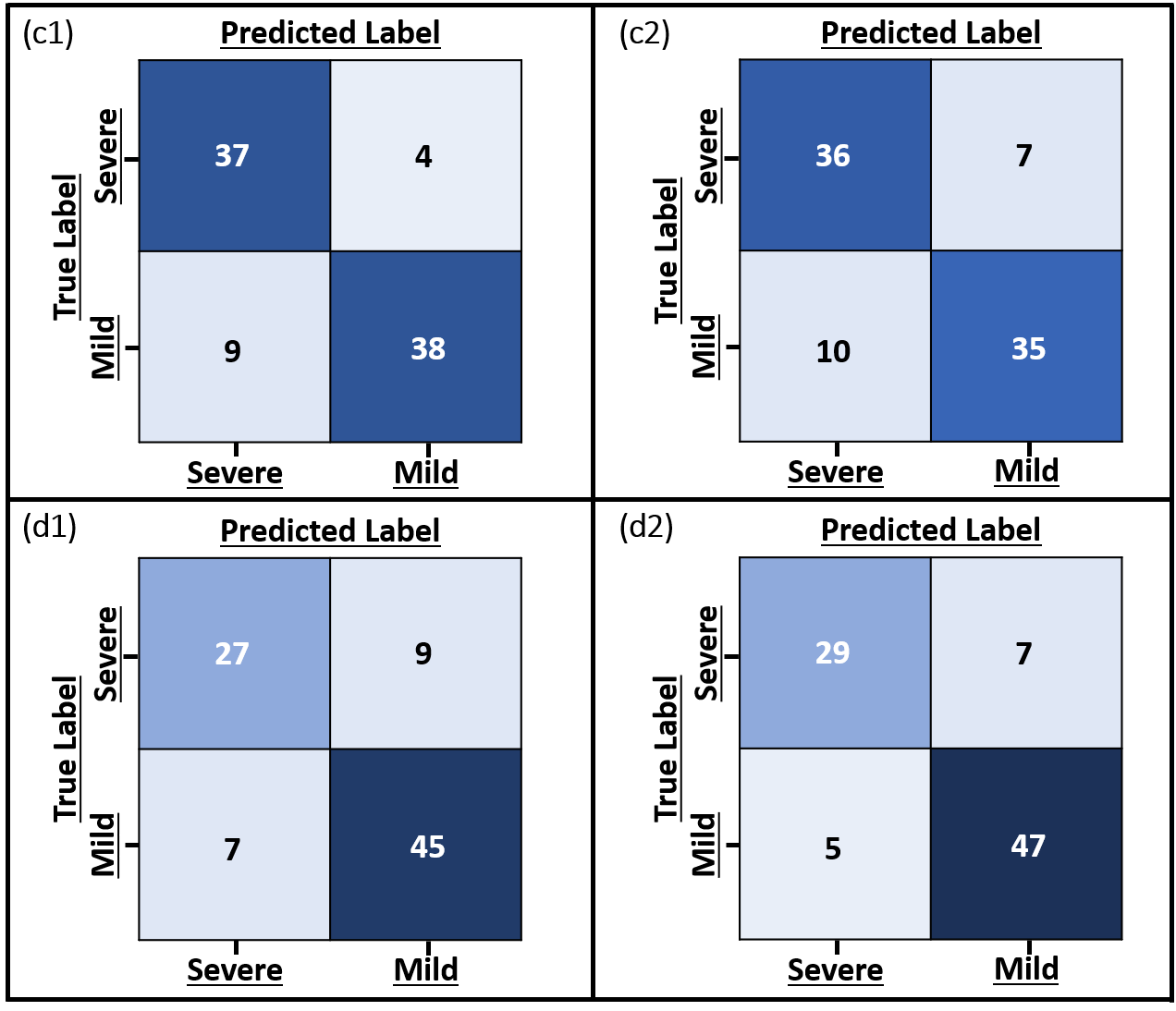


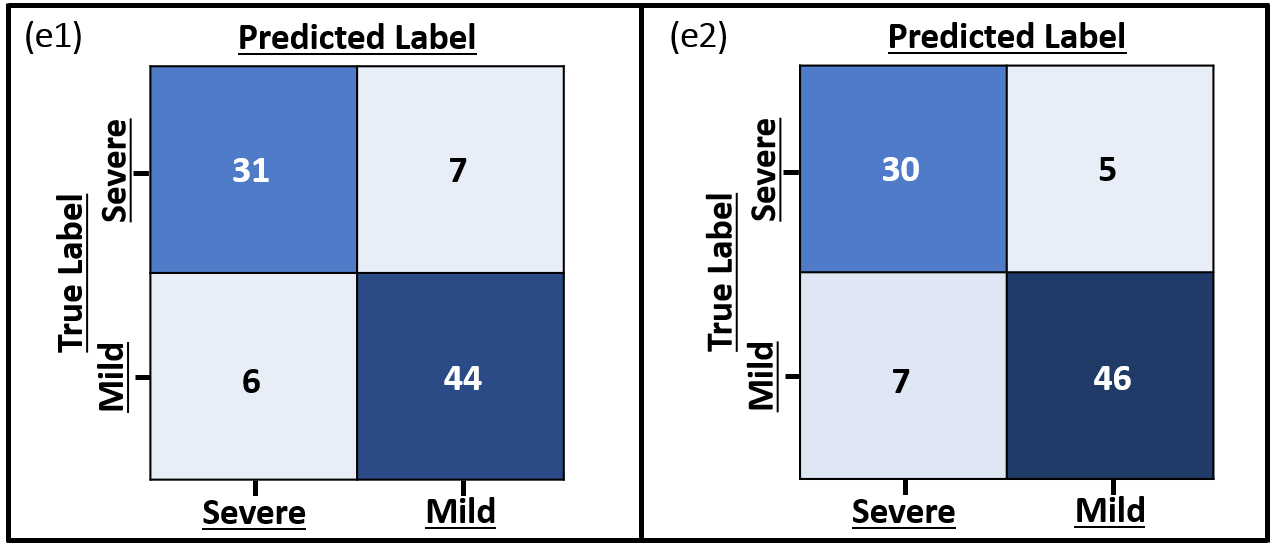


**Figure S4.** Confusion matrix of the trained models for MI-injury sizes using all data for (a1) KNN-1, (a2) SVC-1, (b1) KNN-2, (b2) SVC-2, (c1) KNN-3, (c2) SVC-3, (d1) KNN-4, (d2) SVC-4, (e1) KNN-5, and (e2) SVC-5.
